# The coordinating role of IQGAP1 in the regulation of local, endosome-specific actin networks

**DOI:** 10.1242/bio.022624

**Published:** 2017-04-28

**Authors:** Edward B. Samson, David S. Tsao, Jan Zimak, R. Tyler McLaughlin, Nicholaus J. Trenton, Emily M. Mace, Jordan S. Orange, Volker Schweikhard, Michael R. Diehl

**Affiliations:** 1 Department of Bioengineering, Rice University, Houston, TX 77030, USA; 2 Graduate Program in Systems, Synthetic and Physical Biology, Rice University, Houston, TX 77030, USA; 3 Center for Human Immunobiology, Baylor College of Medicine and Texas Children's Hospital, Houston, TX 77030, USA; 4 Department of Chemistry, Rice University, Houston, TX 77030, USA

**Keywords:** IQGAP1, Cytoskeletal dynamics, Exocyst complex, Membrane processing, Endosome sorting

## Abstract

IQGAP1 is a large, multi-domain scaffold that helps orchestrate cell signaling and cytoskeletal mechanics by controlling interactions among a spectrum of receptors, signaling intermediates, and cytoskeletal proteins. While this coordination is known to impact cell morphology, motility, cell adhesion, and vesicular traffic, among other functions, the spatiotemporal properties and regulatory mechanisms of IQGAP1 have not been fully resolved. Herein, we describe a series of super-resolution and live-cell imaging analyses that identified a role for IQGAP1 in the regulation of an actin cytoskeletal shell surrounding a novel membranous compartment that localizes selectively to the basal cortex of polarized epithelial cells (MCF-10A). We also show that IQGAP1 appears to both stabilize the actin coating and constrain its growth. Loss of compartmental IQGAP1 initiates a disassembly mechanism involving rapid and unconstrained actin polymerization around the compartment and dispersal of its vesicle contents. Together, these findings suggest IQGAP1 achieves this control by harnessing both stabilizing and antagonistic interactions with actin. They also demonstrate the utility of these compartments for image-based investigations of the spatial and temporal dynamics of IQGAP1 within endosome-specific actin networks.

## Abstract (precis)

**Summary:** Super-resolution imaging and multiplexed live-cell analyses indicate the multi-domain scaffold IQGAP1 contributes to a complex regulatory network that coordinates the actin encapsulation and membrane processing of a novel endosomal compartment.

## INTRODUCTION

Many fundamental cellular processes depend on the coordinated activities of membrane and cytoskeletal proteins. The sub-membrane actin cortex of epithelial cells functions as a dynamic filament network that organizes integral membrane proteins and provides structural support that is crucial for the development and maintenance of cell shape, polarity and cell-cell adhesion. Cytoskeletal-dependent membrane tethering, transport, and contractility are also critical to membrane traffic in endocytic pathways (Soldati and Schliwa, 2006; Duleh and Welch, 2010). The cytoskeleton is widely recognized for its role in regulating the motions, local delivery and global spatial distributions of vesicles and organelles within the cytoplasm (Porat-Shliom et al., 2013). Yet, cytoskeletal networks also contribute to membrane and protein traffic at much finer scales, with key examples being the local organelle-specific actin networks that help drive membrane processing and protein sorting mechanisms within specialized endosomal sorting compartments (Muriel et al., 2016; Seaman et al., 2013). These complex sorting stations contribute to numerous cellular processes by controlling the local storage, recycling, and degradation of protein receptors, signaling intermediates, and lipids. They are also increasingly recognized for their ability to function as specialized sensing and signaling platforms that enable local activation or attenuation of signaling via the templating, compartmentalization and physical sequestration of interacting signaling molecules (Murphy et al., 2009; Niehrs, 2012; Villaseñor et al., 2016). The protein machinery that controls membrane scission, vesicle fission/fusion, and intra-vesicle protein and membrane trafficking mechanisms within these compartments has been characterized in significant detail (Jovic et al., 2010; Piper and Katzmann, 2007). Contributions of numerous cytoskeletal proteins and regulatory molecules have also been identified (Anitei and Hoflack, 2012). Nevertheless, the spatio-temporal relationships among these proteins and the associated architecture of the regulatory circuitry that orchestrates the activities of these proteins remain far from fully resolved.

This work examines the role of the IQ motif-containing GTPase activating protein 1 (IQGAP1) in the regulation of a local actin shell that surrounds an endosomal compartment that localizes to the basal cortex of epithelial cells. IQGAP scaffolds are large, multi-domain proteins that function at the interface between cell signaling and cytoskeletal regulation, and have been described as potential coordinators of membrane signaling/dynamics and cytoskeletal regulation in several settings. The best-studied isoform IQGAP1 is expressed ubiquitously and known to participate in a wide range of important biological processes including cell adhesion (Kuroda, 1998), motility (Choi et al., 2013), cytokinesis (Adachi et al., 2014; Bielak-Zmijewska et al., 2008), cell polarization and orientation in tissues (Bañón-Rodríguez et al., 2014), exocytosis (Kimura et al., 2013; Rittmeyer et al., 2008), and phagocytosis (Brandt et al., 2007). The domain organization of IQGAP1 is highly conserved across the IQGAP family. It consists of an amino-terminal calponin homology domain (CHD) followed by six repeats of a 50-amino acid sequence (repeats domain), a WW domain that contains two tryptophan residues, four isoleucine and glutamine IQ motifs, a RasGAP-related (GRD) and a RasGAP C-terminal domain (LeCour et al., 2016). These domains interact with a spectrum of surface membrane receptor, signaling, and cytoskeletal regulatory proteins, and are capable of binding directly with actin filaments (White et al., 2012; Abel et al., 2015).

The multi-domain structure of IQGAP1 should enable multiple modes of actin regulation. Although it does not have GTPase-activating protein (GAP) activity, IQGAP1 binds to and stabilizes the active (GTP-bound) forms of the Rho family GTPases Rac1 and Cdc42 through the GRD domain. These associations have been reported to be sensitive to Ca^2+^/Calmodulin signaling, indicating potential for crosstalk between the functions of the IQ and GRD domains (Watanabe et al., 2015). In addition to these ubiquitous cytoskeletal regulators, IQGAP1 is known to interact with potent actin nucleation/polymerization proteins including the actin-related proteins (Arp) 2/3, which generate branched actin networks, and the Diaphanous-related formin, Dia1, which progressively catalyzes the addition of actin groups to barbed ends through their formin homology (FH) domains (Brandt et al., 2007). Domains in the N-terminal and C-terminal regions of IQGAP1 have also been shown to interact directly with actin filaments *in vitro*. The CHD domain has been reported to bind actin and influence filament-bundling behaviors (Fukata et al., 1997). The C-terminal half of IQGAP1 can also directly cap barbed ends of actin filaments and inhibit actin polymerization (Pelikan-Conchaudron et al., 2011). The identification of these interactions and activities has provided important insights into the regulatory functions of IQGAP1. Nevertheless, despite this success, it is not clear if these activities are mutually exclusive and context-dependent, or whether IQGAP1 can employ multiple actin regulatory interactions within a more complex cytoskeletal regulatory network. Moreover, the challenges associated with visualizing the dynamics of local actin structures and regulatory processes present substantial barriers to characterizing the dynamic spatio-temporal properties of IQGAP1 and its mode(s) of action within local cytoskeletal regulatory networks, limiting abilities to resolve mechanisms describing the coordinating roles IQGAP1 plays in cytomechanical processes.

Herein, we employ a series of high-resolution imaging techniques to examine functional relationships between IQGAP1, actin, and membrane dynamics within a novel endosomal compartment that localizes selectively to the basal cortex of the non-tumorigenic human mammary epithelial cell line, MCF-10A. The compartments contain several IQGAP1 interaction partners and exhibit prominent interactions with recycling endosomes, indicating they function as endocytic sorting stations for adherens junction proteins and other cell surface receptors. High-resolution confocal and super-resolution imaging show that IQGAP1 colocalizes with bundles of actin within the actin shell that surrounds the internal membrane core of the compartments. Live-cell analyses of the hierarchy and timing of IQGAP1, actin, compartment membrane, and membrane proteins indicate IQGAP1 helps regulate the growth, stability and disassembly of the actin shell, and, in turn, the processing and dispersal of its internal vesicular components. We discuss how observations of significant negative correlations between IQGAP1 localization and actin growth at minute timescales, and prominent positive correlations between IQGAP1 and the exocyst complex protein Exo70 across a range of timescales, raise the possibility for a mechanism that relies on the coordinated activities of multiple IQGAP1 domains and highlight the utility of these compartments for imaging-based structural and dynamic analyses of IQGAP1-dependent regulatory networks.

## RESULTS

### IQGAP1 localizes to distinct local compartments in the basal cortex of MCF-10A cells

**Fig. 1.**
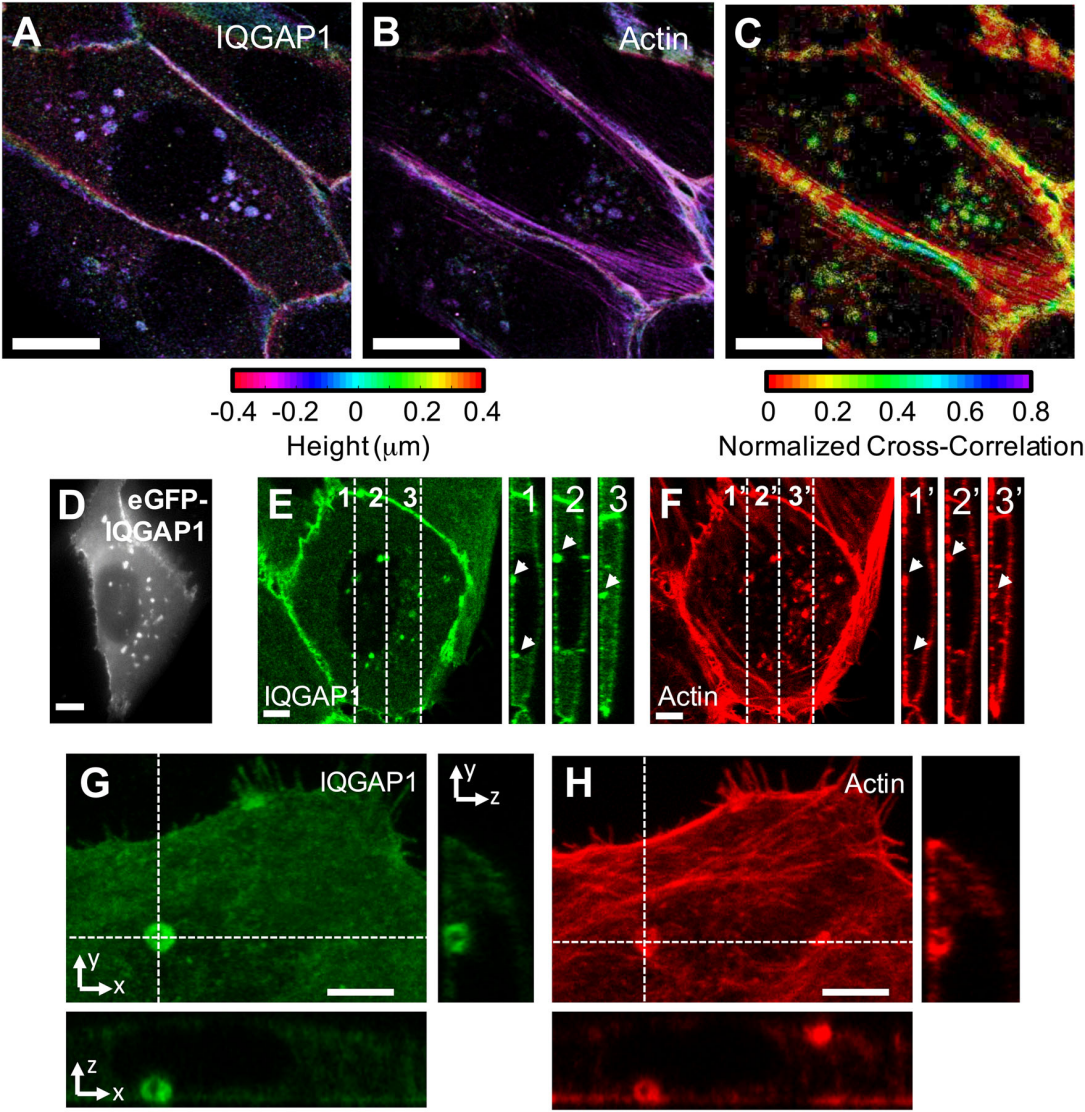
**STORM and confocal images showing IQGAP1 localizes to a specialized actin compartment in the basal cortex of epithelial MCF-10A cells.** 3D STORM images of IQGAP1 (A) and actin (B) acquired via an erasable immunofluorescence imaging procedure and sequential rounds of STORM. The color scale represents axial (*z*)*-*position of the single-molecule centroids. (C) Spatial map of calculated cross-correlation coefficients (*γ*) between IQGAP1 and actin. Similar positive correlations are seen in compartments (*γ*≈0.5) and cell-cell junctions (0.2-0.5). Nearby filamentous actin structures display much lower correlations (*γ* <0.1). (D) Epifluorescence image of a transiently expressed IQGAP1-EGFP construct. (E-H) Confocal images of immuno- and phalloidin-stained IQGAP1 (green) and actin filaments (red). Scale bars for the STORM and confocal images are 10 and 5 μm, respectively. STORM images (A-C) are representative of observations in 15 cells. Additional examples are shown in Fig. S3. Live-cell images (D) represent several hundreds of cells examined in many independent experiments. Confocal images (E-H) are representative images containing 15 cells. Arrowheads in E and F indicate IQGAP1 and actin-positive compartments.

IQGAP1 localization patterns were evaluated in monolayers of MCF-10A cells via a suite of multi-color epifluorescence, confocal and 3D super-resolution imaging procedures (Fig. 1; Fig. S1). In each case, IQGAP1 was found to selectively colocalize at lateral cell-cell junctions with the adherens junction proteins E-cadherin and β-catenin, in addition to actin, as expected (Fig. S1). Super-resolution analyses were performed using stochastic optical reconstruction microscopy (STORM) techniques (Xu et al., 2012). IQGAP1 and actin were imaged sequentially in the same cells via an erasable immunofluorescence staining procedure (Duose et al., 2012; Schweller et al., 2012; Zimak et al., 2012) and consecutive rounds of 3D STORM microscopy. Overall, this approach shows that IQGAP1 co-localizes with a very compact band of actin filaments at cell-cell junctions, but displays little to no associations with other nearby actin structures.

**Fig. 2.**
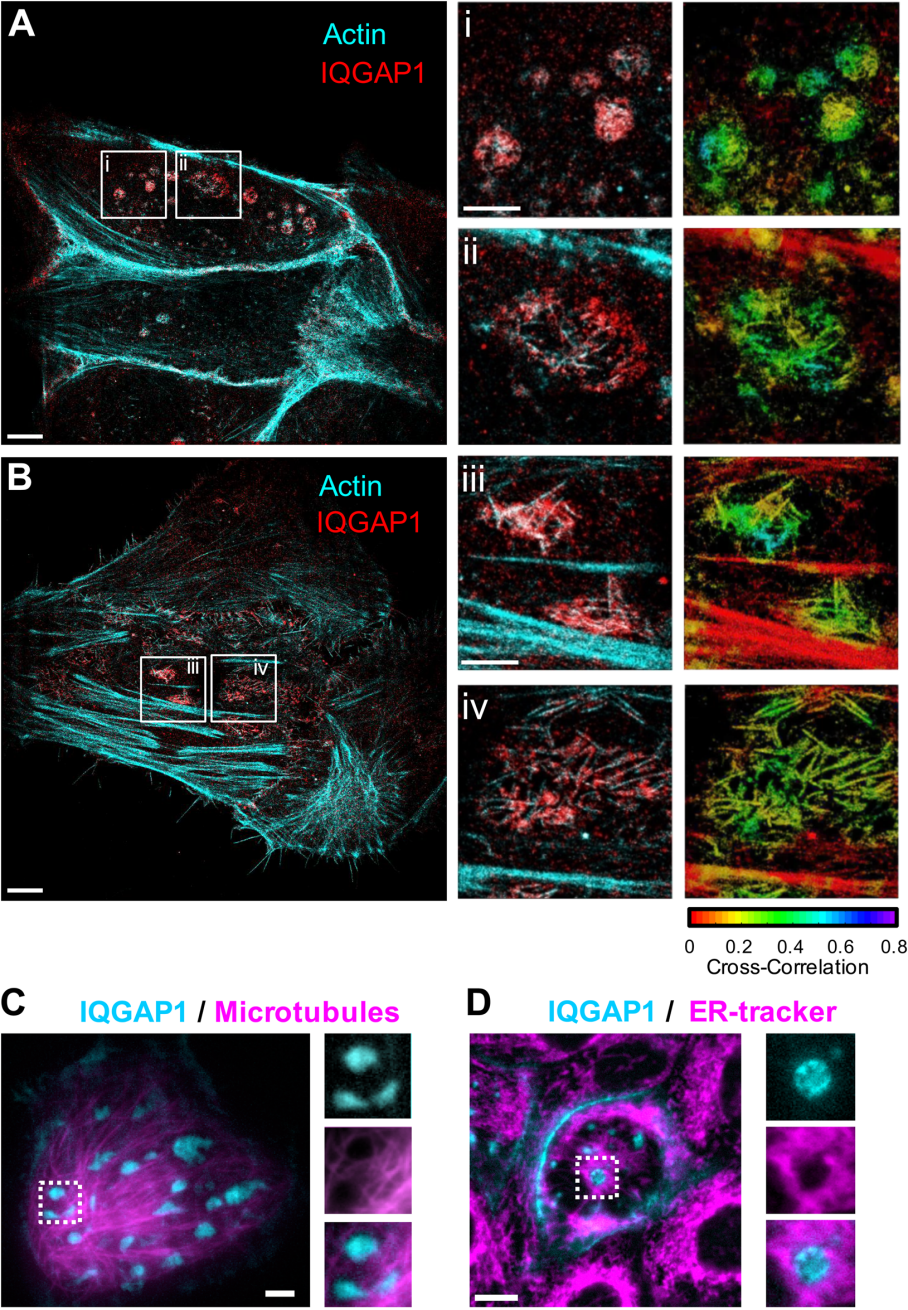
**Structural analyses of the basal IQGAP1/actin compartments.** (A,B) Multiplexed STORM super-resolution images of actin (cyan) and IQGAP1 (red). Insets: Zoomed regions and corresponding image maps of cross correlation coefficients are displayed. (C) Total internal reflection fluorescence (TIRF) image of IQGAP1-YFP and microtubules labeled using an EMTB-mCherry construct incorporating the microtubule binding domain of ensconsin. (D) IQGAP1 compartments are also found to reside in voids of the endoplasmic reticulum stained with ER-tracker Red. STORM images A and B are representative of hundreds of compartments in 15 cells. C and D are representative of 6 and 5 cells, respectively. Scale bars: 5 μm (2 μm for insets).

Epifluorescence imaging, STORM and 3D confocal imaging also all revealed that IQGAP1 localizes to a second actin-containing compartment within the basal cortex of MCF-10A cells (Figs 1 and 2; Figs S1-S3). Large field-of-view epifluorescence images of immunostained endogenous IQGAP1 show that 63% of cells (70/111) have compartments (Fig. S4). The number of compartments per cell is broadly distributed, with a mean (±s.d.) of 3.6±5.9 compartments per cell. Analysis of compartment number in cells stably expressing FusionRed-IQGAP1 showed that compartments were found in 70% (176/270) of cells with a mean (±s.d.) of 4.9±7.2 compartments per cell. The increase in the mean number of compartments per cell with exogenous IQGAP1 expression was not significant (*P*>0.05, Welch's *t*-test).

**Fig. 3.**
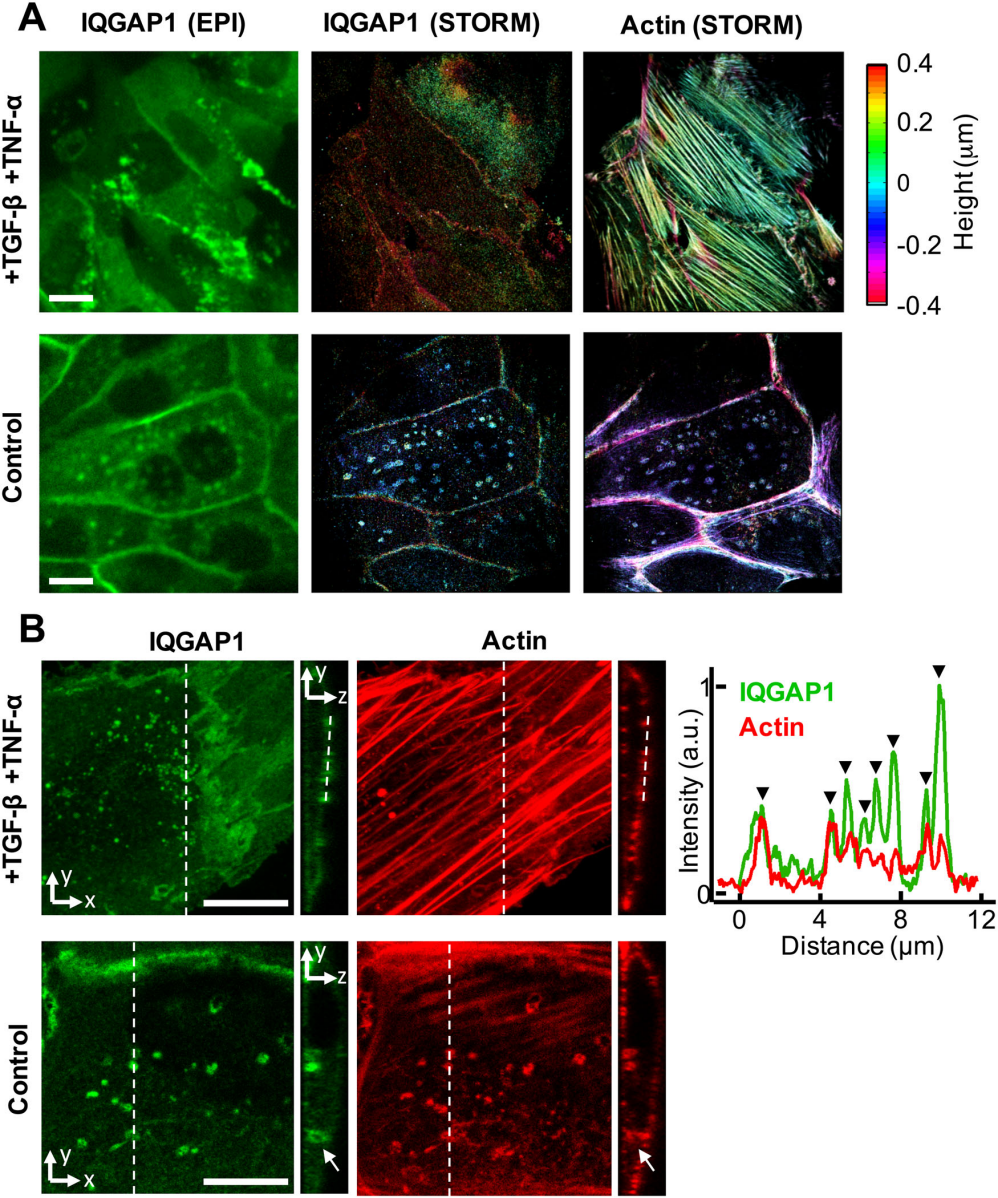
**Basolateral IQGAP1 compartments are unique to polarized epithelial cells.** (A) Epifluorescence and STORM images of cells with disrupted epithelial polarity via incubation with TGF-β1 and TNF-α in cell culture for 24 h (top row) and control cells (bottom row). Basal IQGAP1 structures are not found in treated cells via STORM images (middle column), when used to image a ∼1 μm section near the basal cortex of the cells. (Right column) Pronounced actin stress fibers polarized along the long axis of the cell are specific to cells incubated with TGF-β1 and TNF-α. Scale bars: 10 μm. (B) A 3D confocal image showing that large IQGAP1 compartments are absent in cells incubated with TGF-β1 and TNF-α in cell culture for 24 h (top row); instead, *y-z* slices and corresponding line-intensity plots show that small IQGAP1 puncta that localize to actin stress fibers on the apical cortex are observed, black triangles indicate IQGAP1 puncta. Basal compartments can be found in control cells that were cultured without TGF-β1 and TNF-α (bottom row). For larger compartments it is possible to resolve that IQGAP1 and actin localize specifically to the outer surface of basal compartments (indicated by arrow). Three confocal image stacks were taken for the TGF-β1 TNF-α treated cells, each of which contained numerous apical IQGAP1/actin punctae. Control images representative of 15 epithelial cells in 12 3D confocal images. Scale bars: 10 μm.

Compartment sizes were estimated from epifluorescence image data to avoid the potential effects of sectioning artifacts and to enable surveys of large numbers of cells. These analyses yielded root mean square diameters of 1.18±0.25 µm (mean±s.d.) (Fig. S5). Confocal imaging yielded multiple examples of larger (∼2 µm in diameter) compartments in which it was possible to resolve that IQGAP1 localizes to a hollow actin shell that surrounds the compartment core (Figs 1G,H and 3B). The role of this shell in membrane/endosome traffic is examined below. The STORM images indicate that the actin shell is composed of short and linear filament bundles of actin that appear to be similar in size to the compartments (Fig. 2A,B). Anti-IQGAP1 antibodies were also found to decorate individual filament bundles, indicating IQGAP1 interacts either directly with actin or associates indirectly via specific actin binding proteins. Spatial cross-correlation analyses of IQGAP1 and actin localization at these compartments (see Materials and Methods) reflect this selective association, and notably yield near equivalent cross-correlation values to those found within cell-cell junctions (***γ*** ≈0.2-0.5), indicating that IQGAP1 has a similar propensity to colocalize with actin in both regions.

Finally, live-cell analyses of IQGAP1 compartments indicate that their mobility is highly restricted (Fig. S6, Movie 1). Individual compartments largely remained in place in live cell movies, yielding net sub-diffusive behavior over 30-minute time periods. Their motion appeared to be primarily influenced by whole cell morphological changes. This lack of mobility could be derived from their actin coating. Alternatively, live-cell total internal reflection (TIRF) microscopy also shows that the IQGAP1 compartments tend to occupy local voids within microtubule networks (Fig. 2C; Fig. S7). They also similarly appear in regions that exclude the endoplasmic reticulum (ER) (Fig. 2D; Fig. S7), indicating the microtubule cytoskeleton and the ER may also potentially contribute to their local confinement.

### Basal IQGAP1 compartments are unique to polarized epithelial cells

The basal localization of the compartments indicates that they may either depend on, or even function to support, the apical-basolateral epithelial polarization of the MCF-10A cells. This issue was further investigated by characterizing IQGAP1 trafficking in MCF-10A cells that were driven through an epithelial to mesenchymal (EMT)-like transition by culturing cells in media supplemented with the growth factors TGF-β1 and TNF-α (Fig. 3A; Fig. S8). Immunostained IQGAP1 compartments could not be found in the basal cortex of these cells using TIRF/STORM or confocal microscopy. Instead, small IQGAP1-positive particles appear to associate strongly with stress fibers on the apical side of the cells as a result of EMT. These associations are clearly visible in line-intensity profiles from confocal sections (Fig. 3B). These results indicate the basal localization of the IQGAP1 compartments is unique to the epithelial state.

### IQGAP1 compartments function at the intersection of cadherin junction protein endocytosis and recycling

**Fig. 4.**
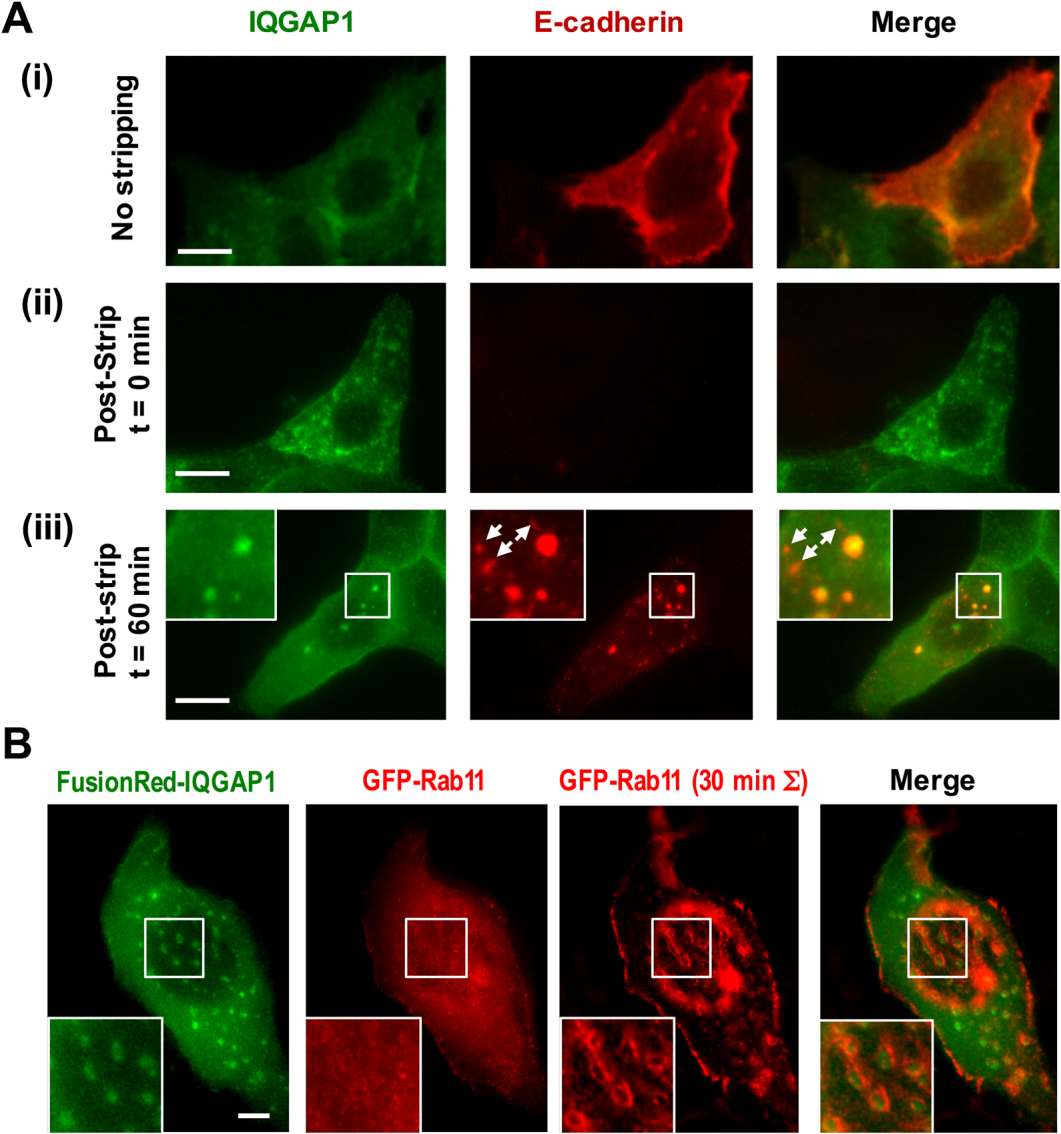
**Basal IQGAP1 compartments participate in E-cadherin endocytosis and recycling.** (A) E-cadherin trafficking was probed via an antibody internalization assay that employs an antibody that targets the extracellular domain of E-cadherin. The top row of images (i) display cells that were incubated with antibodies at 4°C, and then immediately washed to remove unbound antibodies, fixed and stained for immunofluorescence imaging. E-cadherin internalization was examined in rows ii and iii, using cells that were either washed and acid stripped and fixed immediately to remove unbound and surface-bound antibodies (ii), or after 60 min at 37°C to also allow time for receptor internalization (iii). The immunofluorescence images in row iii of the internalized E-cadherin antibodies and IQGAP1 display significant colocalization between the two markers within the basal compartments. After the 60 min incubation period, E-cadherin was found to localize to 18 out of 62 (29%) of compartments in 13 cells. Additional IQGAP1-negative vesicles were also observed (white arrows). (B) Live-cell time-lapse analyses of the transient localization of Rab11-GFP-labeled recycling endosomes at the peripheries of basal compartments labeled with RFP-IQGAP1 (green). Single snapshot images, as well as sums of pixel intensities for Rab11 over 61 images in a movie (30 min) are shown. These images represent movies of eight cells in three independent experiments by two experimenters; an additional example is provided in Movie 2 and Fig. S11. All scale bars: 10 μm.

Considering the known role of IQGAP1 in adherens junction organization and dynamics, we next performed a surface antibody internalization assay (Paterson et al., 2003) to examine whether the compartments participated in E-cadherin trafficking (Fig. 4A; Figs S9 and S10). Anti-E-cadherin antibodies raised against the extracellular domain of E-cadherin were incubated with MCF-10A cells for 1 h at 4°C. The cells were then either washed to remove unbound antibodies, fixed and imaged immediately, or washed, incubated at 37°C to allow for trafficking, and then subsequently acid-stripped to remove the surface-bound antibodies prior to fixation and imaging. The resultant images show that the E-cadherin antibodies traffic to the IQGAP1 positive compartments. The internalized E-cadherin antibodies are also notably found to localize to many additional small, IQGAP1-negative puncta, which we presume are other endosomal compartments that contribute to E-cadherin traffic but do not associate with IQGAP1 (white arrows in Fig. 4A, panel iii). In addition, a separate set of immunofluorescence imaging experiments showed that other adherens junction proteins N-cadherin, β-catenin, and cell surface receptors CD44 also localize to the basal IQGAP1 compartments (Fig. S1). Of interest, E-cadherin, β-catenin, and CD44 were often found within discrete puncta located at the compartment periphery. By contrast, the adhesion receptor CD49f (Integrin α6), did not display strong colocalization with IQGAP1 compartments (Fig. S1).

To further probe the role of the compartments in endocytic pathways, we also examined whether the IQGAP1 compartments co-localized with fluorescent protein constructs that are commonly used as markers to identify early endosomes (Rab5), late endosomes (Rab7), recycling endosomes (Rab11), and autophagosomes (LC3). We also stained for lysosomes and other acidic organelles with the fluorescent dye LysoTracker Red (Fig. 4B; Fig. S11). Finally, we also examined IQGAP1 colocalization with WASH (Fig. S12), an endosome-specific actin regulatory protein that has been shown to regulate endosome shape and traffic (Duleh and Welch, 2010). None of these markers were found to label the IQGAP1 compartments directly. However, time-lapse imaging did reveal significant associations between the basal IQGAP1 compartments and Rab11-positive endosomes (Fig. 4B; Fig. S11, Movie 2). Punctate GFP-Rab11 particles appear to exhibit prominent kiss-and-run interactions. The specificity of these transient interactions is apparent in images constructed by summing pixel intensities from movies, which display clear rings of Rab11 intensity that almost entirely surround the IQGAP1 compartments. Rab11 image contrast at the peripheral membrane also notably increases in the summed images. Both of these behaviors appeared to be unique to Rab11. The remaining Rab and lysosomal markers did not display similar interactions (Movies 3, 4 and 5). While occasional transient associations with Rab5- and Rab7-positive endosomes are observed, it is possible that these associations simply reflect random colocalization events. Negligible associations were also found with LC3-labeled autophagosomes (Fig. S11). Overall, we interpret these data to indicate that, although the IQGAP1 compartments do not present classical endosomal markers directly, they participate in the vesicular trafficking of cell junction proteins and exhibit prominent interactions with recycling endosomes.

### Basal IQGAP1 compartments present multivesicular endosome markers and contain membrane tethering and sculpting machinery

**Fig. 5.**
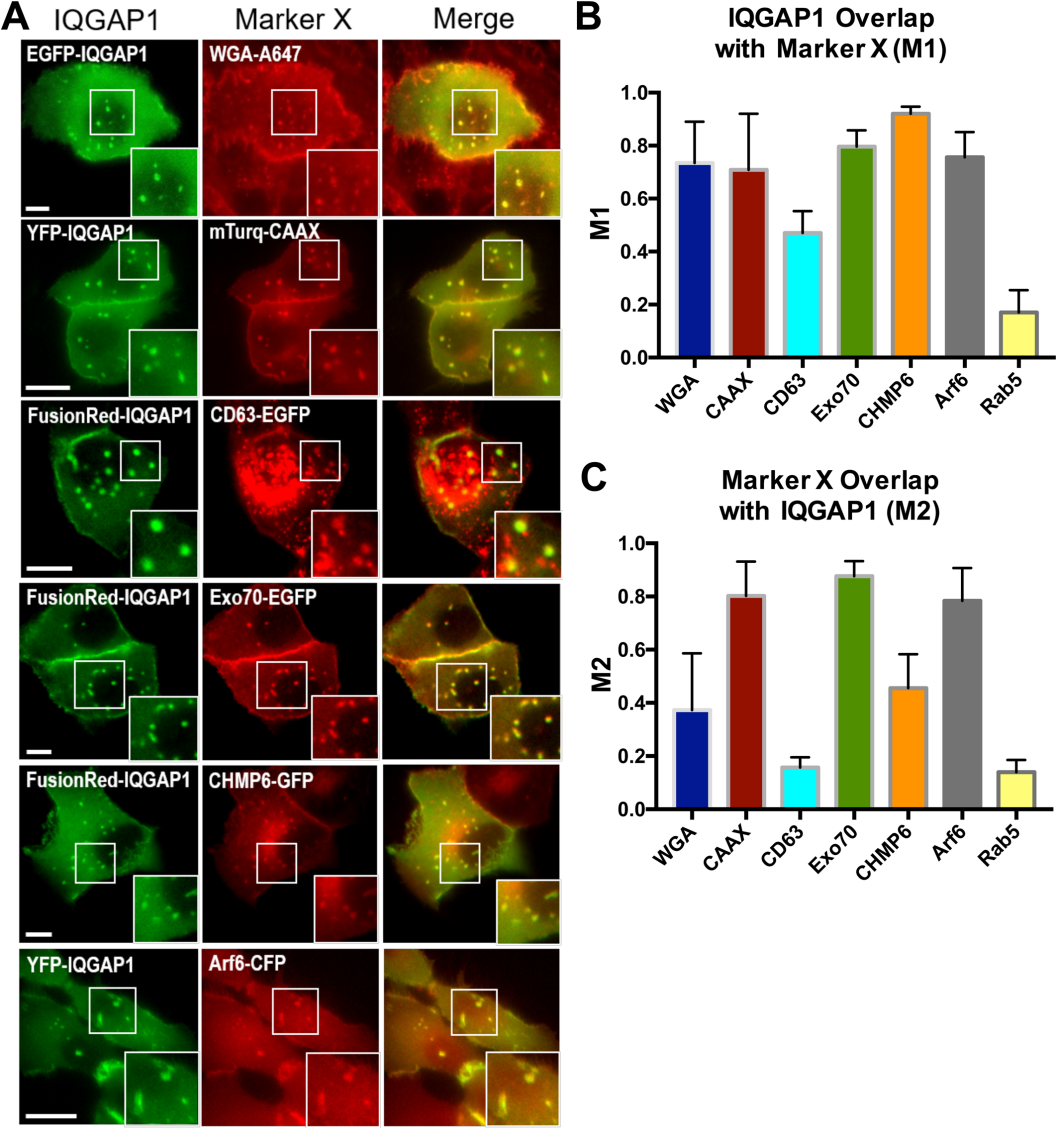
**IQGAP1 compartments present multivesicular endosome markers and contain membrane processing machinery.** (A) Live-cell images show that IQGAP1 compartments colocalize with various multivesicular-endosome-associated proteins. For the live-cell membrane stain, Alexa-647-conjugated WGA was used in place of a transfected construct. Images were processed via 7 pixel rolling ball background subtraction in ImageJ. Scale bars: 10 µm. (B,C) M1 and M2 Manders' colocalization coefficients representing the fraction of IQGAP1 that overlaps with marker X (M1), and fraction of marker X that overlaps with IQGAP1 (M2) (Dunn et al., 2011). Coefficients were calculated using the Coloc2 plugin for ImageJ and averaged across five cells for each marker pairing (error bars=s.d.). Only pixels within the boundary of the cell were considered. Marker expression at cell-cell junctions was excluded; hence, for IQGAP1 a vast majority of the pixels above background were contributed by basal compartments. Statistics for the colocalization analysis are presented in Table S1.

The role of the IQGAP1 compartments in membrane traffic was further investigated via a series of labeling experiments that examined IQGAP1 associations with reagents that stain plasma and endocytosed membrane, classical endosomal markers, as well as membrane-associated proteins that have known roles in endosomal membrane processing and multivesicular endosome dynamics (Fig. 5). Plasma/endocytosed membrane was examined by transfecting MCF-10A cells with an EGFP-IQGAP1 construct and then incubated with Alexa Fluor 647-conjugated wheat germ agglutinin (WGA), a lectin that labels the plasma membrane and vesicles internalized via the endocytic pathway (Cresawn et al., 2007). IQGAP1 was also co-transfected with a YFP-CAAX, that contains the membrane localization sequence of H-Ras (GCMSCKCVLS) to facilitate targeting to the inner plasma-membrane leaflet. Despite the lack of colocalization by endosomal Rab markers described above, both membrane staining methods show significant colocalization with compartmental IQGAP1 (Fig. 5A). Yet, the mTurquoise2-CAAX construct displayed somewhat more selective colocalization within IQGAP1 compared to the WGA, as indicated by calculations of the Manders' colocalization coefficients (Dunn et al., 2011). Here, the M1 coefficient, the fraction of IQGAP1-postive pixels also that also possess positive WGA-Alexa647 or mTurquoise2-CAAX signals are quite similar, whereas the M2 coefficient, the fraction of WGA-Alexa647 or mTurquoise2-CAAX that also display positive IQGAP1 signals differed since more significant overlap is found with the mTurquoise2-CAAX construct (Table S1). This difference appears to stem from the trafficking of the WGA-Alexa647 stain. In this case, substantial numbers of WGA-Alexa647 but IQGAP1-negative vesicles appear to accumulate in the perinuclear space and near the compartments (Movie 6).

We next examined the colocalization of IQGAP1 with several membrane-associated proteins that have known roles in endosomal membrane processing and multivesicular endosome dynamics. IQGAP1 was first co-transfected with a construct containing an enhanced green fluorescent protein (EGFP) gene fusion to the tetraspanin protein CD63 (Fig. 5). Although its precise functions remain undetermined, CD63 is known to be enriched in late endosomes, lysosomes, and exosomes, as well as the intraluminal vesicles of multivesicular bodies (MVBs) (Pols and Klumperman, 2009; Beatty, 2006). The CD63-EGFP construct exhibited diverse punctate staining patterns and was found to localize to both IQGAP1-positive and -negative compartments within the same cells. As a result, the M1 and M2 coefficients followed similar behavior found with the WGA-Alexa647 stain. The CD63-EGFP also tended to be notably dimmer within the IQGAP1 compartments compared to other punctate structures. Much more selective colocalization with surface membrane and compartmental IQGAP1 was found upon co-expression of IQGAP1 with an EGFP-tagged subunit of the exocyst complex, Exo70, an EGFP-labeled construct for charged multivesicular body protein 6 (CHMP6), or a CFP-tagged ADP-ribosylation factor 6 (ARF6) (Fig. 5), particularly for Exo70 and ARF6, which displayed the highest Manders' colocalization coefficients in the series (M1 or M2>0.8). Although additional analyses are likely ultimately required, all of these associations suggest there may be potential roles for membrane sculpting and processing machinery within the compartment. Exo70 has been found to function as a tether for Rab11-positive recycling endosomes at the plasma membrane (Takahashi et al., 2012). It has also been shown to be capable of generating negative membrane curvature presumably via an oligomerization-based mechanism (Zhao et al., 2013). CHMP6 is a key component of the ‘endosomal sorting required for transport’ complex III (ESCRT-III), a complex that plays a central role in the formation and sorting of proteins into MVBs (Henne et al., 2013). ARF6 is a small GTPase that is well known for its roles in membrane processing and signaling (Donaldson, 2003).

### IQGAP1 maintains compartment stability by constraining actin polymerization

**Fig. 6.**
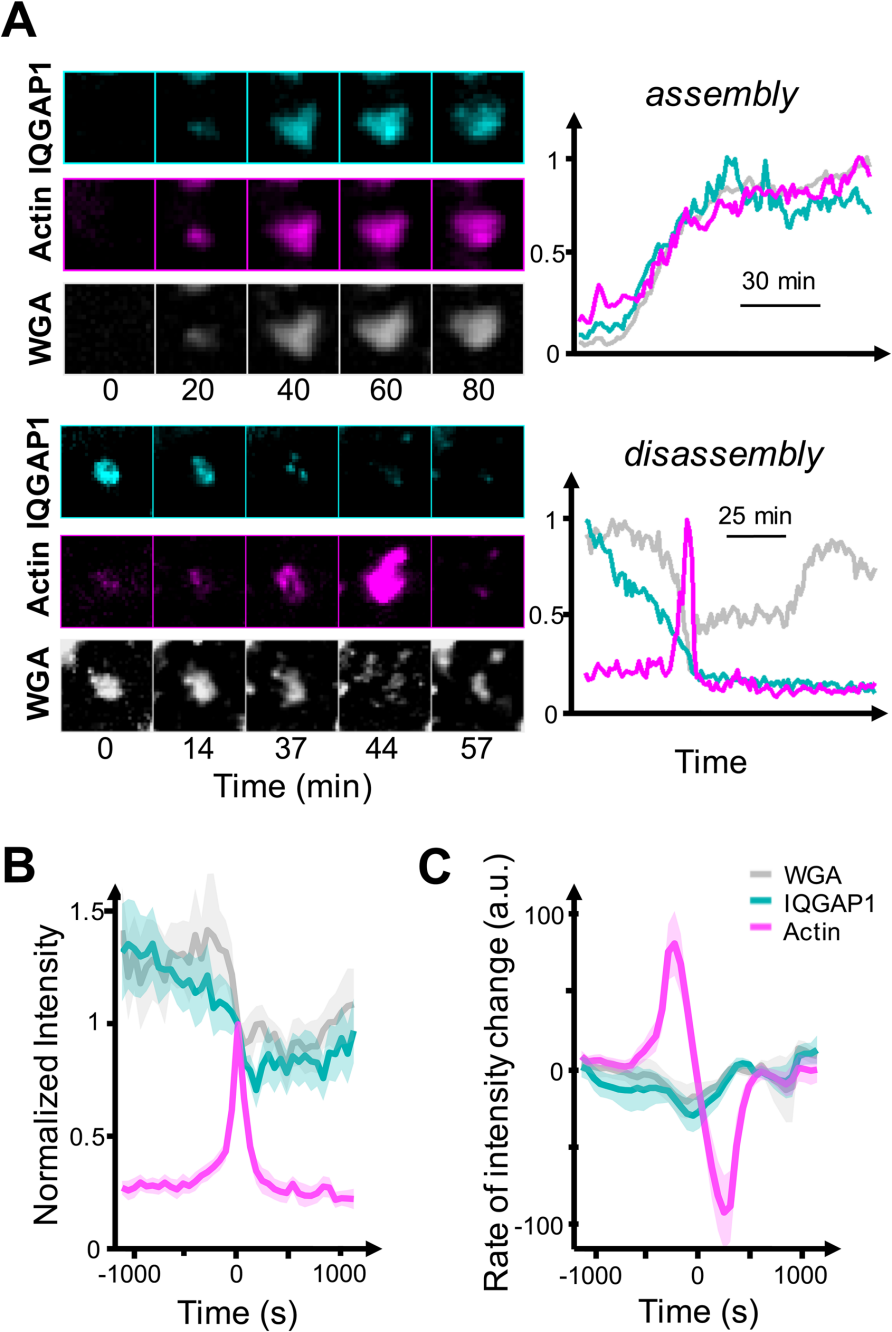
**IQGAP1 and actin dynamics at compartments are coupled with the evolution of the membranous core.** (A) Live-cell analyses of cells co-transfected with F-Tractin-mTurquoise2 and YFP-IQGAP1, and incubated with WGA-A647 membrane stain. Images and corresponding intensity-time traces are displayed for events where compartments assembled and disassembled. Intensities correspond to the summed intensity of all pixels found within a computationally-defined mask that marked compartment boundaries. (B) Plots of average marker intensity as a function of time determined from nine distinct bursting events from five different cells whose trajectories were aligned with respect to the peak of the actin burst, then averaged. Shaded areas represent the mean±s.e.m. (C) Time-derivatives of the averaged traces shown in B. Traces were processed with an 11-point (660 s) Savitzky-Golay low-pass filter before taking the derivative. Negative derivative values for IQGAP1 further confirm its decline prior to the actin burst. Shaded areas represent the mean±s.e.m.

We next performed a series of multi-color live-cell imaging experiments to evaluate dynamic relationships between IQGAP1, F-actin, and WGA-tagged membrane within the basal compartments via transient co-transfection of YFP-IQGAP1, F-Tractin-mTurquoise2, and staining with WGA-Alexa647 (Fig. 6). These experiments showed that the compartments are quite dynamic despite their restricted lateral mobility. Compartments were commonly found to appear and disappear during the course of movies (Movie 1). Significant and rapid IQGAP1 intensity fluctuations were also observed on sub-minute timescales (Movie 7). The lifetimes of the IQGAP1 signals at the compartments also varied considerably and were cell-dependent. The vast majority of compartments remained intact for 90 min or less in some cells, while they appeared to persist over the course of 5-10 h in others (Movie 6). While the mechanistic source of this heterogeneity is difficult to discern at this time, we expect it to be related to differences in metabolic activity and the extent of epithelial polarization.

The time-dependent intensities of YFP-IQGAP1, F-Tractin-mTurquoise2, and WGA were also examined using image segmentation algorithms available in ImageJ to define compartment boundaries. Pixel intensities were then summed to evaluate the temporal dynamics and relative timing of marker trajectories. Compartments were imaged at 30 or 60 s frame rates over periods of 1-2 h since this optimized detection of intensity fluctuations, particularly in the actin channel, while permitting a sufficiently long observation window to visualize compartment assembly and disappearance. IQGAP1, actin and WGA signals were generally found to rise together over a period of approximately 30 min during the assembly of compartments (Fig. 6A, top panel). The most striking IQGAP1-actin-WGA responses, however, occurred during compartment disassembly, which was often preceded by a large burst in actin intensity that lasted a few minutes (Fig. 6A, bottom panel; Fig. S13, Movie 8). The resultant expanded actin shell was found to subsequently depolymerize, disappearing nearly as rapidly as the burst of polymerization. We also observed multiple examples where the central WGA-Alexa647 stained membrane core of the compartment appeared to break up into multiple small vesicles. The resultant smaller vesicles often moved too quickly to be tracked at the present frame rates. Not all actin bursts resulted in compartment disassembly, as similar actin bursts also occurred during periods in between compartment assembly and disassembly (Fig. S13). The dispersion of WGA-Alexa647-positive vesicles was not detected during these events. Yet, all bursting events were preceded by a decrease in IQGAP1 intensity that appears to be sustained throughout the duration of the burst. This temporal relationship is apparent upon visual inspection of individual intensity trajectories, as well as in averages of normalized time-intensity traces where the actin intensity peak during the burst was used to register the busting events in time (Fig. 6B). Time derivatives of these averages also reflect a clear decline in IQGAP1 intensity prior to and during the actin burst (Fig. 6C). In contrast, averaged WGA-Alexa647 signals remained constant on average before and after the burst, while the WGA signals dropped during the bursting event, signifying a loss of membrane components (Fig. 6B).

**Fig. 7.**
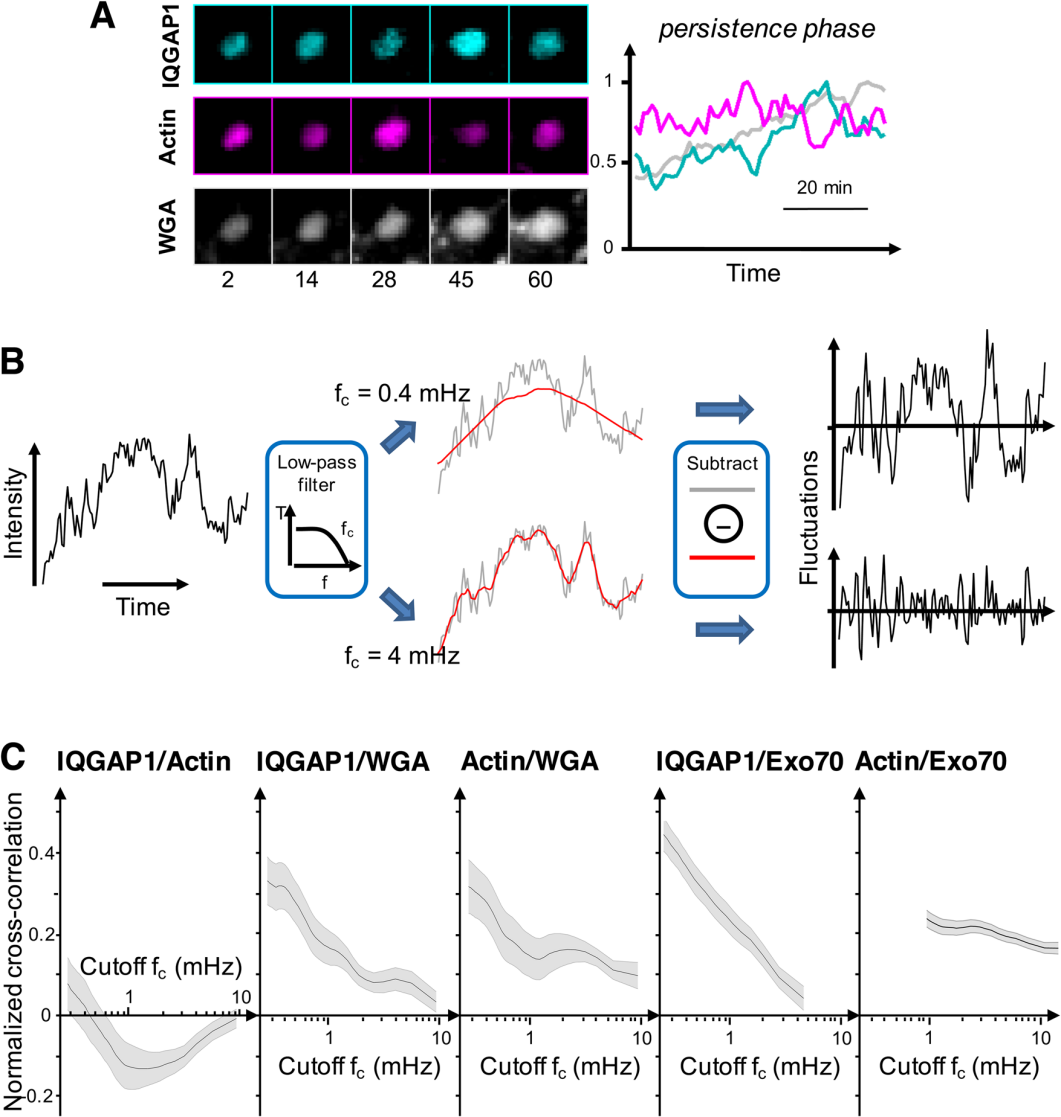
**Temporal correlations between marker intensity fluctuations indicate IQGAP1 may play both protagonistic and antagonistic roles in the dynamics of compartmental actin.** (A) The image-time series and corresponding intensity-time trajectories for IQGAP1, actin and WGA. Anticorrelated intensity fluctuations between IQGAP1 and actin can be seen within the image series and traces. Image data were processed identically to those in Fig. 6. (B) Illustration of the frequency-dependent high-pass filtering to examine temporal correlation among markers, as described in the Materials and methods. (C) The normalized cross-correlations between IQGAP1/Actin, IQGAP1/WGA, Actin/WGA, IQGAP1/Exo70 and Actin/Exo70 using the resulting traces from the procedure in B. Shaded areas represent the average correlation value±s.e.m. (19 compartments from four cells were analyzed for IQGAP1/Actin/WGA, 35 compartments from five cells for IQGAP1/Exo70, and 123 compartments from 11 cells for Actin/Exo70).

These results clearly show that compartment-specific IQGAP1 and actin levels are anti-correlated during actin bursts, and are potentially indicative of an antagonistic interaction between IQGAP1 and actin. To further probe this interaction, we next examined whether IQGAP1 and actin exhibited anti-correlated intensity relationships at other stages in the compartment life cycle. Inspection of time-lapse movies indicated that IQGAP1 and actin intensities fluctuated appreciably when compartments persisted in between assembly and disassembly events (Fig. 7A), and we found various trajectories from multiple cells and independent experiments where IQGAP1 and actin intensities clearly appeared to be anti-correlated during this ‘persistence’ phase (Fig. S13A). While this was not always the case, a quantitative analysis of the temporal correlations between IQGAP1 and actin intensities (Fig. 7) demonstrates that the two markers are indeed anti-correlated on minute timescales on average. Yet, IQGAP1-actin cross-correlations switch from negative to positive values at longer (>1 h) timescales. Overall, the temporal dependence of IQGAP1-actin cross correlation coefficients reflects that, while IQGAP1 and actin fluctuate in an anti-correlated fashion at fast timescales, the intensities of both markers generally grow and shrink with one another on long timescales as the compartments form and disassemble. By contrast, IQGAP1 and WGA intensities, as well as actin and WGA intensity, correlate positively at all timescales. We interpret the latter behaviors to mean that IQGAP1 recruitment may stabilize the compartment, potentially by crosslinking and stabilizing the filament network. Yet, IQGAP1 also appears to play a second, concurrent antagonistic role that restricts local actin polymerization around the compartment. Together, these behaviors appear to contribute to a regulatory mechanism where compartment assembly and disassembly appears to be controlled by momentary losses of IQGAP1 and transient bursts of actin polymerization.

### Timing of Exo70, CHMP6 and IQGAP1 signals

**Fig. 8.**
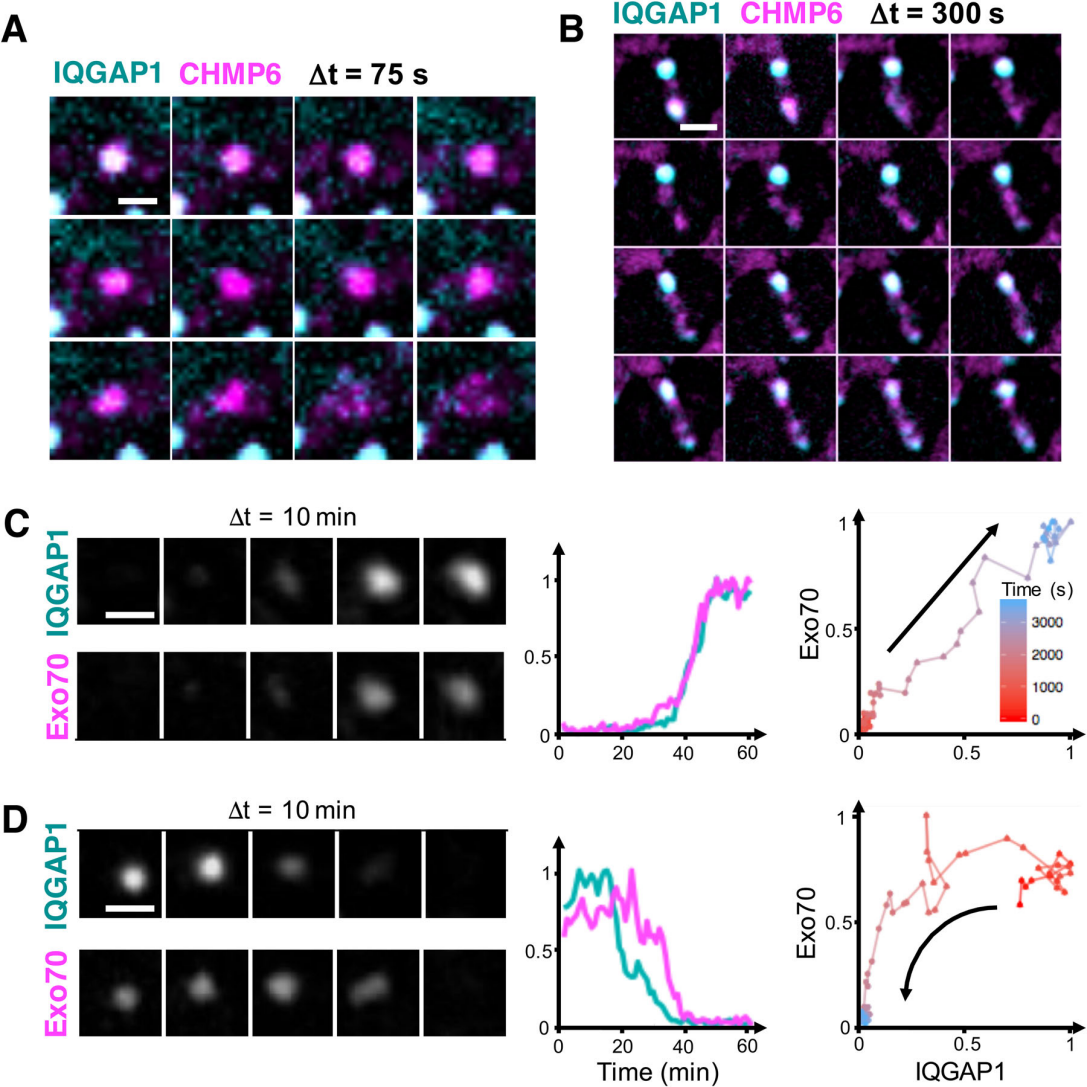
**IQGAP1 and Exo70 and CHMP6 localize and dissociate to and from compartments via distinct mechanisms.** (A,B) Images from a movie showing a CHMP6-GFP construct is retained on highly motile vesicles that are released upon dissociation of FusionRed-IQGAP1 (compartment disassembly). The images in B also show the trafficking of CHMP6-positive vesicles to a neighboring IQGAP1-positive compartment, and a slow re-acquisition of IQGAP1 around CHMP6-positive vesicles that remain near the original compartment. (C) MCF-10A cells were co-transfected with FusionRed-IQGAP1 and EGFP-Exo70. Movie frames and corresponding marker intensity traces during compartment assembly show that IQGAP1 and Exo70 intensities rise proportionally, yielding a linear relationship when IQGAP1 signal is plotted versus Exo70. (D) Loss of IQGAP1 precedes that of Exo70 during compartment disassembly, resulting in downward concave curvature in IQGAP1 versus Exo70 intensity plots. Plots for C and D were generated from 14 traces/5 cells, and 15 traces/3 cells, respectively. Scale bars: 2 μm (for A, C and D) and 10 μm (for B).

We next examined the temporal relationships between IQGAP1 and the membrane-associated proteins Exo70 and CHMP6. Live-cell imaging shows that the ESCRT III complex protein CHMP6 was retained on compartment vesicles even after disassociation of IQGAP1 (Fig. 8A). Moreover, similarly to movies of the WGA membrane stain, multiple small, CHMP6-positive vesicles appeared to be released from the compartment in these instances (Movie 9). We also found examples where a subset of CHMP6-bearing vesicles was trafficked to a different IQGAP1-positive compartment, while other vesicles from the original compartment appeared to re-acquire a new IQGAP1 signal (Fig. 8B).

Finally, to gain insights into IQGAP1-membrane interactions we performed cross-correlation analyses of IQGAP1 and Exo70 intensities (Fig. 7C). In contrast to IQGAP1 actin trends, these two markers are strongly positively correlated on long timescales, with a decreasing trend towards faster timescales. However, correlation plots of IQGAP1 versus Exo70 intensities indicate the associations underlying these correlations depend on whether compartments are assembling or are in the process of disassembling. Here, time-dependent intensities of Exo70 were generally found to increase linearly with increasing IQGAP1 signals during compartment assembly (Fig. 8C). However, Exo70 tended to dissociate either with or after IQGAP1 during compartment disassembly (Fig. 8D), with near equal frequency (7/14 events versus 6/14 events, respectively). In the latter cases, IQGAP1 intensities decreased faster than Exo70 levels, yielding non-linear, downward concave curvature when Exo70 is plotted as a function of IQGAP1 intensity.

## DISCUSSION

The multi-domain scaffold IQGAP1 is recognized increasingly for its abilities to function at key nodes within signaling pathways and orchestrate the activities of membrane receptors, both membrane and intracellular signaling molecules and cytoskeletal proteins (Choi et al., 2016; Jacquemet et al., 2013). Yet, despite numerous examples of roles in local cytoskeletal mechanics, the subcellular spatial and temporal dynamics of IQGAP1 have not been investigated in significant detail, limiting abilities to construct mechanistic pictures describing how local signaling and cytoskeletal dynamics are coordinated within and around these structures. This study outlines the discovery that IQGAP1 helps regulate the assembly and disassembly of an actin shell that selectively surrounds an endosomal compartment that localizes to the basal cortex of MCF-10A epithelial cells. Immunofluorescence and live-cell imaging demonstrate that multiple membrane markers, membrane receptors and membrane trafficking proteins associate with IQGAP1 within these compartments, many of which are known to interact with IQGAP1 in other subcellular settings. We interpret these data to indicate that the compartments function as local endosomal sorting stations and likely participate in membrane and/or membrane protein recycling. Notable behaviors reflecting this function include IQGAP1 colocalization with the exocyst complex component, Exo70 and the GTPase ARF6, and observations of prominent associations/kissing interactions between the compartments and Rab11-positive recycling endosomes. Overall, the basal localization, restricted mobility and relatively short life-cycle of these compartments, along with the strong actin signals and ability to visualize multiple compartments simultaneously within individual cells, provide unique opportunities to characterize local IQGAP1 and actin dynamics via multiple precision microscopy approaches. We generally find that these properties minimize potential ‘interference’ by the non-local/background actin dynamics within the rest of the cell, alleviating issues that can confound spatio-temporal analyses of organelle-specific actin networks in other cellular settings.

While epifluorescence and high-resolution confocal imaging indicate that actin forms a shell that surrounds the central membrane core of the compartments, super-resolution image analyses using STORM demonstrate that IQGAP1 associates with bundled actin filaments within this shell. This organization is quite different from the discrete patches of actin that have been shown to assemble on certain classes of endosomes via Arp2/3-WASH-dependent mechanisms (Derivery et al., 2009; Seaman et al., 2013). These local, endosome-specific networks have been proposed to provide a structural framework to assemble local signaling networks. They presumably also help facilitate local force generation during the production or scission of endosomal tubules. While it is certainly possible that the actin shell of the present endosomal compartments also contributes to local signaling and membrane mechanics, we believe our data is consistent with a protective function, where the actin shell both confines the membrane components to the basal actin cortex and shields them from interactions with other endosomal compartments, as we found a number of instances where multiple small endosomes emerge from the compartment and are dispersed rapidly upon actin shell disassembly.

Interestingly, dynamic live-cell imaging data also provides evidence that IQGAP1 regulates the stability of the actin shell by harnessing a combination of supportive and antagonistic interactions with actin. Fluorescent signals from IQGAP1, actin, and WGA-stained membrane were found to rise together on average as the compartments are assembled. IQGAP1 was also retained until the compartments began to disassemble and their internal membrane vesicles were released. However, surprisingly, compartment disassembly involved the rapid and seemingly unconstrained growth of the actin shell. These brief events were preceded by a significant decrease in the total compartmental IQGAP1 signal. While similar actin bursting events were also observed that did not appear to be associated with complete compartment disassembly, IQGAP1 intensities decreased prior to these events as well. Moreover, we found many instances where IQGAP1 and actin fluctuations appeared to be negatively correlated within compartment intensity trajectories and IQGAP1 and actin cross correlation coefficients were negative at intermediate timescales. Together, these observations and the IQGAP1 decoration of actin bundles in STORM images support a regulatory mechanism where IQGAP1 simultaneously plays both a stabilizing role to help maintain the shell integrity and an inhibitory role to restrict actin polymerization.

A next operative question will be how IQGAP1 coordinates the activities of its multiple domains to exhibit such complex and multifaceted behavior. Prior analyses of IQGAP1 actin interactions provide some clues. While the N-terminal CHD of IQGAP1 has been shown to bind actin, and multiple domains including the CHD, IQ and GRD domains have been proposed to support IQGAP1 dimerization/multimerization under certain conditions (LeCour et al., 2016; Liu et al., 2016; Ren et al., 2005); although this behavior remains controversial (Nouri et al., 2016). Nevertheless, IQGAP1 dimerization or higher-order multimerization could potentially support actin crosslinking, bundling and stabilization of the actin shell. There is also evidence that IQGAP1 recruitment could potentially inhibit further actin polymerization within the shell. The C-terminus of IQGAP1 has been shown to function as an actin capping protein and to block actin growth *in vitro* by preferentially binding ADP-actin subunits at the barbed end of filaments (Pelikan-Conchaudron et al., 2011). IQGAP is also known to function as an important regulator of Rho family GTPases through its GRD domain (Fukata et al., 1997; Watanabe et al., 2015). These associations could also potentially inhibit the functions of actin nucleation promoting factors and elongation proteins. In either case, controlled growth of the actin shell could then be achieved via momentary loss of IQGAP1 to permit actin polymerization, and then reacquisition of IQGAP1 to re-stabilize the structure. More significant and sustained loss of IQGAP1 could similarly result in the large actin bursts during compartment disassembly. It is interesting to note that this general model framework resembles a negative feedback network where actin growth recruits IQGAP1 to inhibit further actin growth.

Finally, our observations that IQGAP1 and exo70 localization correlate positively over a range of timescales highlights the possibility that IQGAP1 helps coordinate actin and membrane dynamics within the compartment. IQGAP1 has been reported to interact with the exocyst complex to help drive vesicle tethering in invadopodia during tumor cell invasion downstream of GTPase signaling (Sakurai-Yageta et al., 2008). The N-terminus of IQGAP1 has also been shown to bind the exocyst/septin complex and enhance polarized secretion in pancreatic β-cells (Rittmeyer et al., 2008). In this case, over-expression of an IQGAP1 C-terminal domain containing the GRD domain was found to inhibit secretion, indicating a link to GTPase signaling. It is therefore quite possible that IQGAP1 operates within a broader regulatory circuit to coordinate exocyst-dependent membrane processing events with the dynamic growth and disassembly of the actin shell. IQGAP1 has also been demonstrated to interact with other significant membrane tethering and signaling proteins (Choi et al., 2016). These interactions and the potential for IQGAP1 to occupy multiple functional states within the endosomal compartments must certainly be considered. Nevertheless, we believe that resolving mechanistic pictures of the complex regulatory framework will ultimately require abilities to relate the dynamic functions of IQGAP1 domains to those activities of actin and membrane regulatory proteins. We also anticipate the endosomal compartments will constitute a useful platform for these analyses.

## MATERIALS AND METHODS

### Cell culture and fixation

MCF-10A human mammary gland cells were purchased from ATCC (CRL-10317) a cultured in unfiltered MEGM without GA-1000 (Lonza, TX, USA) supplemented with penicillin, streptomycin, and ampicillin, and 100 ng/ml cholera toxin, unless noted otherwise. Cells were trypsinized and then seeded onto fibronectin- or matrigel-coated coverslips at partial confluency and incubated for a period of 24-72 h. Cell fixation was carried out using 4% paraformaldehyde in MEGM that did not contain GA-1000 and bovine pituitary extract. Afterwards, cells were washed with phosphate buffered saline (PBS) and incubated in a solution of NaBH_4_ for 7 min to quench the fixation reaction. After a final wash with PBS cells were permeabilized with 0.5% triton-x 100 for 10 min.

Antibody staining procedures were performed as follows. Samples were incubated in blocking buffer (BB) composed of Herring sperm DNA, bovine serum albumin (BSA), polyT-DNA, dextran, ampicillin, and NaN_3_, in PBS for at least 15 min. Primary antibodies were diluted in a PBS solution containing 10% BB. Mouse primaries for β-catenin (Santa Cruz Biotechnology, CA, USA; SC-7963), E-cadherin (Santa Cruz Biotechnology, CA, USA; SC-8426), N-Cadherin (BD Pharmigen, CA, USA; 561553), CD44 (BD Biosciences, CA, USA; BDB550392), and CD49f (BD Pharmingen, CA, USA; 555734) were incubated overnight, at room temperature at a final concentration of 2 µg/ml. Mouse anti-ɑ-tubulin (Sigma Aldrich, MO, USA; T6199-200ul) and rabbit anti-IQGAP1 (Santa Cruz Biotechnology, CA, USA; SC-10792) antibodies were incubated in blocking buffer for 1-2 h at 37°C at a concentration of 2 µg/ml. Selectivity of the rabbit IQGAP1 antibody was tested via co-labeling with an exogenously expressed mYFP-IQGAP1 construct (Fig. S2). Cells were washed three times with PBS to remove unbound antibodies, and then incubated with secondary antibodies in blocking buffer at 37°C. IQGAP1 primaries were stained using goat anti-rabbit Cy3 (Life Technologies; A10520). All other primary antibodies were stained using goat anti-mouse Cy5 (Life Technologies; A10524). The samples were finally rinsed three times in a PBS solution prior to imaging. Note that an alternative clone was tested to confirm selective IQGAP1 localization to the compartments (Fig. S2).

### Cell labeling and gene nucleofection and for live-cell analyses

Live-cell stains for the ER, lysosomes, and lipid membranes (ER-Tracker Red, LysoTracker Red and WGA Alexa-647, respectively) were obtained from Molecular Probes (OR, USA). Plasmids encoding for genes of the following were purchased from Addgene (MA, USA): GFP-Rab11 WT (plasmid #12674), DsRed-Rab7 WT (plasmid #12661) (Choudhury et al., 2002), mCherry-Rab5 (plasmid #49201) (Friedman et al., 2010) EMTB-mCherry (plasmid # 26742) (Miller and Bement, 2009), CHMP6-GFP (plasmid # 31806) (Guizetti et al., 2011), EGFP-Exo70 (plasmid # 53761) (Martin et al., 2014), CD63-pEGFP C2 (plasmid # 62964), EGFP-IQGAP1 (plasmid # 30112) (Ren et al., 2005), FusionRed-Lifeact (plasmid # 54778) (Shemiakina et al., 2012). The IQGAP1 and F-tractin structural genes were amplified by high-fidelity PCR to generate expression constructs with different fluorescent protein dyes, permitting multicolor microscopy with the different marker constructs listed above. The resultant gene fusions were inserted into custom-designed vectors that were engineered to support their subsequent assembly into larger, multiple gene vectors as described by Guye et al. (2013).

Cells were passaged 48-72 h before the day of nucleofecting. For one sample, 0.5×10^6^ cells were pelleted and resuspended in 100 µl of supplemented Nucleofector Solution T (Lonza, Texas, USA). 100 µl of this mixture was added to approximately 2 µg of plasmid DNA. For transfections with two constructs, 1.5 µg of each was used. Samples were electroporated using a Nucleofector 2b device (Lonza, TX, USA) set to program T-024. Nucleofected cells were resuspended immediately in pre-warmed, complete MEGM and allowed to recover overnight before being seeded onto coverslips coated with Growth Factor Reduced Matrigel (Corning, NY, USA). Live-cell imaging was conducted 24 h after seeding.

### Epifluorescence and confocal microscopy

Epifluorescence images were acquired using a Nikon Eclipse Ti-E microscope using 60× NA 1.4 oil, 40× NA 0.95 air and 20× NA 0.5 air objectives and a 14-bit depth EMCCD (Andor Technology, Belfast, Northern Ireland). Images are presented without any processing, except for the following images, which were background subtracted with a 7 pixel rolling ball algorithm in ImageJ (NIH): Figs 2D and 4B (‘30 min ∑’ and Rab11 channel in ‘Merge’ image only), Figs 6, 7 and 8, Figs S11 and S12 (‘Sum’ panels), and Movies 2-6, 8 and 9, and lastly, Fig. S5 for which background subtraction was performed in MATLAB (Mathworks, MA, USA) as described in the caption.

All confocal imaging was performed using a Leica SP8 laser scanning confocal microscope with a 100×1.4 NA objective. Multi-channel images were acquired by sequential scanning. Data was acquired with LASAF software and exported to ImageJ for analysis.

### Time-lapse analyses of compartment dynamics

Time traces of marker intensities (IQGAP1, Actin, WGA) within a compartment were generated using image segmentation algorithms available in ImageJ to define the boundaries of compartments, and thus, image masks that could be used for subsequent intensity analyses. Masks were generated for each frame of time-lapse movies, and then merged into a final larger mask to allow changes in compartment intensity to be monitored with minimal influence from compartment motions during the course of the movies. Time-intensity trajectories were generated by summing pixel intensity values within the final mask for each movie frame.

To quantify the cross-correlations between fast intensity fluctuations of each pair of markers, time traces were high-pass filtered by subtracting a trace that was processed with a Savitzky-Golay low-pass filter. Normalized cross-correlation coefficients (*χ*) between the filtered traces were computed for each marker pair as a function of the filter cutoff frequency. Values were then plotted with respect to the 3 dB frequency of the filter.

### Multi-round STORM imaging

STORM imaging was performed using a custom-built inverted TIRF microscope. A single 642 nm laser (DL640-100, Crystalaser, NV, USA) was used to activate, deactivate, and excite fluorescence. The laser was focused at the back focal plane of a high numerical aperture objective to achieve a collimated sample illumination of 2 kW cm^−2^. An oblique excitation scheme was used to reduce fluorescence background and limit the laser illumination to the first few microns beyond the glass/water interface formed by the coverslip and the sample. Fluorescent emissions were collected by the same objective (Nikon, Tokyo, Japan, Plan Apo TIRF 60×, oil immersion, 1.45 NA) and separated from the illumination light by a multi-band dichroic mirror and band pass filter. A 400 mm achromatic lens was used to image the filtered emission onto an EMCCD camera (DU-897E-CS0-#BV, Andor Imaging, Belfast, UK). Three-dimensional imaging was achieved by placing a weak cylindrical lens (f=1 m) into the emission pathway just before the EMCCCD camera (Huang et al., 2008). All STORM imaging experiments were conducted using commercially available coverslips containing embedded gold nanoparticles (Hestzig, VA, USA). The point spread function (PSF) from the stable emissions of these nanoparticles provided fiducial markers for the alignment of STORM reconstructions from each round using custom MATLAB software.

Cells for STORM analyses were fixed and stained with the rabbit IQGAP1 primary antibodies as described in Schweller et al. (2012), Zimak et al. (2012) and Duose et al. (2012) with the modifications outlined in Fig. S3.

The antibody cross-correlation maps in Figs 1 and 2 were generated as follows. Sequentially acquired IQGAP1 and actin STORM images were aligned to one another using custom MATLAB software that either maximized the overall cross-correlation between the two images or aligned the centroids of emissions from fiducial gold nanoparticles. MATLAB software was then used to generate a two-dimensional normalized cross correlation heat map, where the heat map value at pixel *u*,*v* represents the normalized cross correlation *γ*(*u*,*v*) between IQGAP1 and actin localizations, averaged within a 580 nm radius from pixel *u,v*. Values for *γ*(*u*,*v*) were calculated via the following equation:

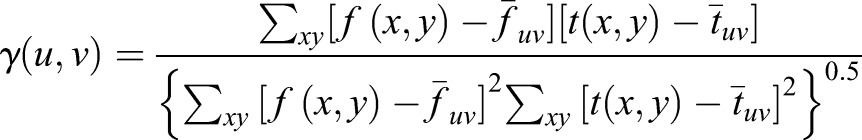
where *f*(*x*,*y*) and *t*(*x*,*y*) represent the total number of binned localizations at pixel *x*,*y* of the actin and IQGAP1 source images, respectively, and the summation is over all pixels *x*,*y* that are within a set radius (580 nm) of pixel *u,v*. 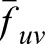 and 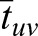 represent mean number of binned localizations for the subset of pixels *x*,*y* in the actin and IQGAP1 channels, respectively.

### E-cadherin internalization assay

A surface labeling strategy, originally developed to follow transferrin receptor internalization, was employed to probe the internalization of surface-bound E-cadherin (Paterson et al., 2003). Cells were passaged 24 h prior to experiments and seeded to yield sub-confluent layers. Initially surface-bound E-cadherin was labeled with monoclonal antibody HECD-1, directed against the extracellular domain of E-cadherin, by incubating cells for 1 h at 4°C in Hanks' balanced salt solution including 1.26 mM Ca and 0.89 mM Mg (HBSS+Ca+Mg) +50 mg/ml bovine serum albumin (BSA) +5 μg/ml HECD1 antibody. Cells were then washed with PBS to remove any remaining unbound antibody, and transferred to HBSS+Ca+Mg at 37°C to allow for internalization of surface-labeled E-cadherin. After varying amounts of time, cells were washed with 0.5 M Acetic acid +0.5 M NaCl (3×5′ washes) to strip any residual antibodies remaining on the cell surface. Cells were then fixed, permeabilized and stained with fluorescent secondary antibody to detect the internalized E-cadherin. Samples were also stained to visualize IQGAP1 via immunofluorescence.
